# Nobody Dies Alone in the Electronic Patient Record—A Qualitative Analysis of the Textual Practices of Documenting Dying and Death

**DOI:** 10.1177/00302228211019197

**Published:** 2021-05-26

**Authors:** Laila Hov, Bodil Tveit, Oddgeir Synnes

**Affiliations:** 87368 VID Specialized University, Oslo, Norway

**Keywords:** communication, death, attitudes death, caregivers, hospitals

## Abstract

In this study, we analyse the electronic patient record (EPR) as a genre and investigate how a death is documented as part of the EPR, that is, what kind of textual practices can be found, and how they can be understood based on extracts from 42 EPRs from medical wards in Norwegian hospitals. Following from our analysis, we see four distinct patterns in the documentation of patient death: a) registering the bare minimum of information, b) registering a body stopped working, c) documenting dying quietly and placing it in peaceful surroundings, and d) highlighting the accompanied death. The textual practices of documenting the transition to death in the EPR make death appear manageable and sanitised, depicting death as either uneventful or good. While the EPR genre is steeped in biomedical language, other discourses relating to death can be seen as ways to accommodate the ideal of a dignified death.

Medical texts in a hospital context, whether they are admission notes, discharge summaries, shift notes, referrals, treatment plans, care plans, or expert assessments, are fundamental elements in the scheme of specialist healthcare. In today’s hospital, these forms and notes are a part of the electronic patient record (EPR), thus making the EPR a powerful communication tool in constant and direct dialogue with the ongoing everyday practices of healthcare professionals. Furthermore, electronic patient records communicate with the patients and next of kin (Schryer, 1993). Thus, EPRs serve a dual purpose—to maintain a record of healthcare events and act as a legal document that is potentially available to patients or families.

In a recent news story regarding a complaint about the treatment and care of a patient in a Norwegian hospital, attention was drawn to the documentation in the EPR and that its representation of the patient’s death did not correspond with the family’s experience. The family observed that the patient suffered severely in his last minutes and was “tormented to death.” The EPR, on the other hand, described that the patient became quiet at the end and “went to sleep” (a Norwegian lay-term and common expression for dying, our translation). The family’s version was supported by the Norwegian Board of Health Supervision (Rostad, 2020).

This story points out that EPRs do not provide an objective textual representation of what “really happened”; instead, they are a significant social actor that produces its own reality and thus aids in achieving expected effects (Hov et al., 2020a; Aarseth et al., 2017). Inspired by Engebretsen (2005) and Andersen’s (2002) application of Bakhtin’s notion of dialogic language, we see the utterances made in an EPR as consisting of a dialogical interplay between *living voices* (healthcare professionals, patients, and family), rather than anonymous utterances unattributed to a source. In line with Engebretsen, we argue that this documentation carries an ethical responsibility to make the voices of others (e.g., the dying patient and their next of kin) heard (Engebretsen, 2005) and that, in these notes, the patients are particularly vulnerable.

Hospital documentation of the transition from living to dead takes place in the context of bureaucratic institutions, in which treatment and care reflect the limitations and constraints of the organisation rather than (only) the needs of individual patients (Walter, 2017, p. 18). Additionally, in contemporary medicine, death is often viewed in terms of failure or giving up (Chapple, 2010; Kaufman, 2005; Lakasing, 2014; Walter, 2017), which may contribute to an understanding of death as something that can be defeated or at least “always” postponed (Kaufman, 2010; Lakasing, 2014; Walter, 2017). Researchers problematise that contemporary dying is being hidden away in institutions, medicalised and alienated from society and our everyday lives (Aries, 1981; Kartzow, 2019; Kellehear, 2016; Walter, 2017). Furthermore, research suggests that professionals experience a lack of opportunity to work through the deaths of their patients, making this line of work more demanding (Ådland et al., 2019; Hopkinson et al., 2005; Ramvi & Gripsrud, 2017; Zheng et al., 2018).

In writing the EPR, the author must attend to several issues: providing an accurate representation of the patient’s case (Pasientjournalforskriften, 2019); fulfilling the document’s organisational role as a communication tool, using the “correct” language and verifying that legal rules, regulations and guidelines were followed. To ensure that the document *works as intended*, the writing must comply with the (unwritten) rules and practices of documentation. By unwritten rules we refer to the established ways of writing that is shared by the healthcare professionals, without being incorporated as formal demands or obligations. These (unwritten) rules and practices influence how the texts are molded by the writers’ understanding of the expectations of colleagues and the organization (Garfinkel, 2009). Poirier (2004) describes medical texts as highly professional, aiming to present selected information “efficiently and consistently” (p. 57). Through the choice of words and phrases, the EPR text also conveys values, although these values are not necessarily those of the writer but rather values based on the shared understanding of how and what is perceived as relevant to document (Engebretsen, 2007). In line with the values of modern medicine, medical texts value objectivity, neutrality, efficiency and the verification of a job correctly performed (Berg, 1996; Hurwitz, 2017).

In this study, we will investigate the transition to death as a part of the EPR as a genre (Schryer, 1993). Traditionally, “genre” is used to distinguish between literary variations based on their characteristic features (e.g., novels vs. poems) (Bakhtin, 1998). Unlike literary texts, Pomata (2014) argues, medical texts are part of an epistemic genre that has evolved in relation to science because their main purpose is to transmit knowledge about a patient and treatment rather than producing meaning as such. Another relevant aspect of medical texts as a genre is found in Kennedy’s analysis of medical cases from patients with heart failure in the nineteenth century (Kennedy, 2014). She discovered that although medical realism and clinical discourses are ubiquitous in medical texts, the genre sometimes “displays a range of discursive approaches beyond the clinical by expressing empathy towards the patient’s condition” (p. 106).

Previous research on the textual representation of death in EPRs is scant, but a notable exception is Habeck-Fardy (2019), who executed an audit of the terminology of death in discharge summaries and discovered that “irrespective of clinician level, doctors use euphemisms to convey death.” This is analogous to Aaslestad’s investigation of psychiatric records as textual practices over100-year period, from which he concluded that, at all times, the journal text remained silent about difficult topics. He also pointed out how the journal text more-or-less reflects the contemporary discourses and understandings of diseases (Aaslestad, 2016).

In the present study, we investigated how death is communicated through the EPR genre. We do so with a theoretical lens rooted in Bakhtin’s notion of genre and language as a dialogic interaction in which reciprocity and responsibility emerge between at least two poles in the exchange of utterances (Andersen, 2002; Bakhtin, 1998; Bakhtin et al., 2010). Bakhtin states that utterances only make sense when they are uttered as a response to a previous utterance or they anticipate a particular response from others (Andersen, 2002; Bakhtin, 1998; Bakhtin et al., 2010). Echoing Bakhtin, Schryer defines genres as relatively stable ideological structures that “embody the unexamined or tacit way of performing some social action” (Schryer, 1993, p. 209). This makes an analysis of EPR texts useful in gaining insights into how medical writing is shaped by “certain expectations the community of writers and readers develop based on previous experience with similar texts” (Pethes, 2014, p. 25). Bakhtin divides utterances into two categories, dialogic or monologic, based on the utterances’ *openness* to other voices (Martin & White, 2007). Martin and White further elaborated on the concept of dialogism and argued that utterances are neither monologic nor dialogic but rather exist on a continuum between these two extremes.

The present article is part of a larger study on the EPRs of patients who died in Norwegian hospitals. In the first article (Hov et al., 2020a), we investigated how physicians negotiate the turning point from curative to palliative care in the days leading up to death. In the second article (Hov et al., 2020b), we investigated how numbering, timing and categorising impact the documentation of the patient’s last 24 hours of life. In this study, we ask the following questions: how is the moment of death documented, what kind of textual practices can be found, and how can they be understood as part of the EPR genre?

## Methods and Materials

### Data Collection

The data for this study were collected from hospitals in primary healthcare facilities in Norway. Excerpts of 42 EPRs from eleven medical wards were collected. Data collection took place between March and June of 2017 in accordance with the following inclusion criteria: a) the patient was over 18 years of age at the time of death, and b) the discharge summary stated that death was an anticipated outcome. The selected records belonged to the most recently deceased patients in each participating hospital. The documentation was anonymised before the data were handed over to the researchers.

The included hospitals used an EPR system known as a distributed information and patient data system for hospitals (DIPS, Norwegian acronym) (DIPS, 2020), the most common system for EPRs in Norwegian hospitals. In DIPS, each group of health professionals has access to a designated template, depending on their profession. Death was recorded in three documents: nurses’ shift notes, physicians’ journal notes, and physicians’ discharge summaries.

### Materials

The youngest person was in her early thirties, and the oldest was around 100 years old; mainly, patients were above 75 years old. The most common causes of death reported in the discharge summary were cancer, organ failure, stroke, and infections such as pneumonia or sepsis.

In the 42 EPRs, the transition from living to dead was documented a total of 81 times—33 times in nurses’ shift notes, eight times in physicians’ journal notes, and 40 times in physicians’ discharge summaries), implying that in nine EPRs, nurses had not documented the death. In two cases, the physician documented the death only in journal notes, meaning that no discharge summary was written. In six cases, the death was documented twice by the physician—first in journal notes and then in the discharge summary. A common feature of these six cases is that death occurred unexpectedly, or the physician was summoned shortly before its occurrence. All cases involved at least one documentation of the death.

### Data Analysis

We used qualitative text analysis, where we explored the documentation of the transition to death informed by Bakhtin’s notion of genre. The basis for the notion of genre is Bakhtin’s understanding of language as dialogic. The dialogic language is rooted in the idea that communication can only be possible, have meaning and make sense in a dialogical interplay between a “me” and a “you” (Dentith, 2004; Andersen, 2002).

According to Bakhtin, utterances (both written and spoken) are directed towards a specific area of utilization or sphere of speech, and this shapes and influence what and how the utterance is shaped (Bakhtin, 1998, p 1). One specific sphere of communication makes up a genre and can be understood as “a typical form of utterances” in a sphere of communication (Andersen, 2002, p. 85). The presumption that language is dialogic.

This implies an understanding of the medical texts as productive contributors to the (re)production of the ongoing dialogue that is in constant negotiation with the relatively stable EPR genre (Andersen, 2002; Bakhtin, 1998; Class, 2014; Patton, 2014). We executed an analysis of the selected notes, searching for content and linguistic representations with which to identify tendencies and patterns in the text, as well as omissions. The first tendency we noticed was a pattern of rephrasing the transition to death using euphemisms describing death as “going to sleep”. This recurrent rephrasing of death as sleep was unexpected, which prompted us to map out the various patterns in which dying was documented across hospitals and professional affiliations.

In subsequent work, we carried out a phrase-by-phrase analysis of the notes and their orientation toward the EPR genre, choice of words, contextual framing of death, deviations from the patterns and emotional expressions. We grouped the emerging features into themes pertinent to our research question and theoretical framework (Prior, 2013). Two researchers on the team (LH and BT) are healthcare professionals (nurses), and therefore, they experienced in both reading and writing medical records in clinical practice.

### Ethical Considerations

The medical record is a sensitive source of information that is reserved for healthcare professionals and the patient. Requesting access to the documentation of deceased patients required careful preparation. Ensuring patient anonymity was our main concern. The regional ethics committee of Norway granted the project a limited exemption from confidentiality standards, allowing one person employed at the included hospitals to retrieve and anonymise medical records for the project (2016/2035/REK). Data protection officers at the hospitals approved the project before our contacts began selecting EPRs from medical wards that were not directly associated with palliative care patients.

The anonymisation implied removal of all proper names, ages, dates, and diagnosis codes. Additionally, the names of the authors were removed, but information about their professional rank was retained. Age was reported in 5-year ranges, and the person retrieving the material was asked to include gender and whether the patient was admitted from home, a nursing home or a transfer from another hospital.

## Results

We found four distinct patterns in documenting death in the EPR: a) registering the bare minimum of information, b) registering a body stopped working, c) documenting dying quietly and placing it in peaceful surroundings, and d) highlighting the accompanied death. Many notes included elements of more than one of these patterns.

The topics that nurses and physicians document vary according to their professional approach to the dying patient, although the ways in which different professions document the transition to death have a great deal in common; thus, we chose to present the results under the same heading and identify the writers’ profession in brackets after the example. In regard to the documentation of death, the commonly used terms equate death with sleep: “to go to sleep” (å sovne inn) or “to go to sleep quietly” (“å sovnestille inn”). We also found variations of the word “go” (“gå” in Norwegian) used as a common rephrasing of death. Here, we have used the words “to leave” in the translation to English as an equivalent euphemism in that language.

### Registering the Bare Minimum of Information

A common tendency in documenting the transition to death was registering the bare minimum of information:

MORS (date) at 22:50. (Physician, discharge summary, Case 1)

The patient died (date) at 09:50. (Physician, discharge summary, Case 2)

Pt. died at 06:15. Undersigned and next-of-kin were present when it happened. (Nurse, shift note, Case 3)

The patient went to sleep around 20:00 the (date). Expected MORS. (Physician, discharge summary, Case 4)

By documenting the transition to death in this way, most circumstantial details were removed, making the note more similar to the way death is documented on death certificates. Apart from the time of death, we learned that the family was present in Case 3 and that death was expected in Case 4.In two examples, we see the Latin word for death, “MORS”, in uppercase letters. Why the word is written in uppercase letters is not clear. Perhaps emphasising the word makes the communication more efficient, and thus, the message will be passed along more quickly. However, “MORS” is the only word written in uppercase letters in the notes. In the last example, we also noticed that the word death is replaced by “sleep”. This way of writing concurs with the common way of documenting other tasks and events in the material—efficiently with limited vocabulary, focusing on passing the message.

### A Body Stopped Working

The most dominant tendency in documenting the transition from living to dead is a description of a body ceasing to function:

Telemetry shows gradual brief atrial fibrillation, gradually increasing bundle branch block pattern, and gradual ventricular replacement rhythm. Finally, asystolic at 17:37. (Physician, journal note, Case 5)

Reporting of respiration ceased at 14:25. (Physician, journal note, Case 6)

Undersigned checked on the patient at 22:45. The patient had rapid respiration and no gurgling. The patient was calm, with no signs of pain. The patient was not conscious. The patient’s girlfriend was sleeping next to him. A bit after 23:00 a colleague was there, the patient had rapid respiration. At around 23:30, a colleague was there; the patient did not have any respiration. (Nurse, shift note, Case 7)

In the two physician notes, the reporting only concerns body functions. In the nurse note, we are taken into the course of events, which are presented as a timeline. These examples depict death as biomedical, occurring through the cessation of either heartbeat or breathing.

Another tendency that also depicts death as a failing body is describing how the body stopped responding to treatment:

Clinical- and laboratory-wise pneumonia, treated with (antibiotics) with good biochemical effect. Despite this, the patient became clinically worsened after admission and died peacefully (date). (Physician, discharge summary, Case 8)

Unfortunately, poor clinical and chemical response. The patient died on (date) with next-of-kin present. (Physician, discharge note, Case 9)

In this way of documentation, the text indicates that the death occurs due to the patient’s body failing to respond to the professionals’ attempts to save the patient.

### Dying Quietly in Peaceful Surroundings

As the two previous tendencies reveal, notes on the transition to death are efficient and have a biomedical orientation. Thus, we were surprised to discover that, in almost one-third of the cases, the word “death” was replaced by “sleep” or “leave”, and, in several cases, the text placed the transition to death in peaceful surroundings:

Pt went to sleep quietly and calmly with next-of-kin present at 12:02. (Nurse, shift note, Case 10)

Pt. had calm respiration at the beginning of the shift. Became considerably dyspneic and gurgling during morning care. Received morphine x1. Was aspirated x1, a lot of thick mucus. More and more strained respiration. Fetched next-of-kin. Pt took her last four breaths and went to sleep quiet and calmly at 09:00. (Nurse, shift note, Case 11)

Informed over telephone by nurse who says that the patient died at 14:43 in bed under peaceful surroundings and with spouse present. (Physician, journal note, Case 12)

Has not been responsive during this shift. Does not seem to have pain. Leaves calmly and quietly ad mortem at 04:00. (Nurse, shift note, Case 13)

The patient died under peaceful circumstances with his girlfriend and family around him. (Physician, discharge summary, Case 7)

The patient departed in peaceful surroundings at (time). (Physician, discharge summary, Case 14)

By using paraphrasing to connote sleep or leaving, together with adjectives such as “peacefully” or “quietly” and sometimes describing the circumstances and surroundings, the backdrop of a busy hospital ward is replaced by serenity, transcending the biomedical representation of death. This embellishment of death occurred just as frequently in nurses’ as in physicians’ documentation, so it does not appear to be linked to profession.

Case 11 differs from the other cases in that the record more explicitly communicates a difficult death:

When we come into his room, we find him unresponsive, no heart or lung sound. Has a lot of green regular vomit in bed. No traces of blood. Looks like he has had a cardiac arrest some minutes before we arrived. Declare him dead around 18:00. (Physician, journal note, Case 15)

However, the visual details presented in the journal were removed from the discharge summary:

The patient dies later in the evening when he is found unresponsive. Declared dead at 18:30 (date). Family informed. Rest in peace. (Physician, discharge summary, Case 15)

In this version of death, the vomit is removed, and a final farewell from physician to patient is added with the phrase “rest in peace”. This is the only example of a remark addressed to the patient.

### Highlighting the Accompanied Death—Silencing the Lonely Death

The documentation of the family being present seems to be a priority for healthcare professionals. In most cases, the presence of another person was thematised in the text by either the nurse or the physician:

The condition worsened after 18:00, and patient went to sleep with family present. (Nurse, shift note, Case 9)

The patient died peacefully with family present. (Physician, discharge summary, Case 16)

In cases in which time is scarce, efforts are made to ensure that the family will arrive in time:

Family arrives after the physician has called for them. We call once more and ask them to run. They make it just in time for the patient’s last breath. (Nurse, shift note, Case 17)

The importance of not dying alone is underscored at the patient’s death bed in cases in which the family is not able to be there or when there is no family.

Pt has, at undersigned’s arrival, gone to sleep with nurse by his side. It all went fast, and the patient was without pain. (Physician, journal note, Case 18)

A change in respiration from 21:30, shallower. Increasing sallowness and pallor in the face. Went to sleep 20:50 with nurse present. (Nurse, shift note, Case 1)

In six of the 42 cases, there is no report of either family or nurse being present at the time of death, but rather than stating that the patient was alone, the text is silent on the absence of another person.

On arrival to the ward (date), she is not conscious. Pt. dies the (date) of severe heart failure and gastro-intestinal bleeding. (Physician, discharge summary, Case 19)

Only in case 15 is there an explicit indication of the patient being alone at the time of death, stressing that he or she was not left alone for long: “Looks like he had a cardiac arrest some minutes before we arrived.” However, the word “alone” was never used in the notes.

## Discussion

To our knowledge, this study is the first to explore the textual practices of documenting the transition to death. Using the concept of genre (Bakhtin, 1998; Class, 2014; Schryer, 1993) as an analytical lens, we reveal that EPR notes contain four patterns, which we found across professions, wards, and hospitals.

### A Genre That Makes Death Manageable, Uneventful and Sanitised

As a reader, we rarely encounter any smells, sounds, agony, suffering, and disturbing aspects of dying described in the notes. The transition to death is recorded in terms of the time of death, a body that stopped working, a task failed (the body did not respond to the professionals’ efforts to save the patient), and/or calm or quiet surroundings. Avoiding or not explicitly describing that death can be brutal or uncomfortable is a clear tendency; instead, death is sanitised and made into a manageable task, primarily a biomedical event.

Kellehear problematises the way in which the “seduction of medical rescue” (Kellehear, 2016, p. 11) contributes to narrative in both medicine and society, in which medicines and technologies are offered as the solution to all issues in healthcare, including death (Chapple, 2010;Kaufman, 2005). This induces society at large to focus on the likelihood that healthcare crises will be resolved by medical interventions, and “dying becomes a medical event rather than a natural event in which medicine plays a part” (Walter, 2017, p. 18). Previous research on genre has shown that the medical genre is shaped by current views of medicine and society (Kennedy, 2014; Aaslestad, 2016). We argue that this medicalisation (Walter, 2017) also influences the documentation of dying, a consequence of which is the alienation of human suffering. In the notes, dying appears to be simplified as either uneventful, mechanical/task-oriented or dignified, with little in between, and the documentation of death contributes to maintaining a discourse on dying as a work task, a biological event in which a body stopped working. It is not depicted as an existential issue.

### A Porous Genre: Discourses From Society and a “Good” Death Keep Seeping in

Although the transition to death was often written about in a neutral, objective and efficient manner, we frequently came across traces of discourses that are more common in society at large. One example is the lay terms for death that enter the genre, with the most common being “gone to sleep”, which present the transition to death as dignified, comfortable, “good” and in line with how society prefers death to occur. In today’s society, having a good death is increasingly challenging because dying is denied recognition until shortly before death (Kellehear, 2016; Hov et al., 2020a,b) and when it is finally recognised, it often becomes medically determined within an institutional context. Essential to the idea of a good death is the recognition of death as a complex process that often takes time (Kellehear, 2016). Therefore, we wonder what the connotation of a good death communicates. What does it mean to die in peaceful surroundings when the patient is treated with curative aims until mere hours before death? As far back as 1968, Glaser and Strauss (1968) used the term “postmortem story” to describe how the death of a patient triggers “a social-psychological process that brings the story of the patient’s dying and death to a close in their [healthcare professionals’] minds” (p.230). Also, in a study of nursing staff’s work with deceased patients, Ådland et al. (2019) concluded that the “rites of passage in the transition from life to death are a necessary part of care for the patient because the nursing home staff clearly struggled to make sense of their experience with the dead body” (p. 9). However, Ramvi and Gripsrud (2017) addresses how talk about death is silenced in healthcare institutions and the lack of space in which healthcare professionals can talk about the loss of patients.

Our assumption is not that the EPR can or should serve a function of closure for healthcare professionals, but we acknowledge that these notes transform a potentially emotional experience into words. It is feasible that genre rules affect the process of writing notes by making it too much of a genre breach to address potentially difficult feelings/experiences. The EPR genre is not adapted to the unveiling of any emotional distress the professional may have experienced in their work with the patient, making it easier to maintain professional distance. As we see it, Case 15 is probably the only case in which we notice a resistance to the typical genre pattern of leaving out the difficult elements—first, in the physician’s visual description of the dead patient and then, in the words “*rest in peace*” in the discharge summary.

Another aspect of the EPR connoting a good death relates to the accompanied death. As pointed out above, dying in the presence of another person is perhaps the most consistent pattern in the documentation, and there is no suggestion that the patient might wish it otherwise. The need to document the presence of another person is strong in modern society—an accompanied death is preferred to dying alone (Kellehear, 2009). There is a clear conception that dying is easier when it is shared with others (Kellehear, 2016), and this has eclipsed the “ability to see this group [the dying] in pluralist terms” (Kellehear, 2009, p. 5). This implies that if a patient wishes to die alone, it may be perceived as unthinkable for the professional, so when it happens, it may be experienced as an unsuccessful death by the professionals, thus resulting in a need to hide its *aloneness*, which is in line with Aaslestad’s analysis of handling difficult topics with silence (Aaslestad, 2016).

### The Ethical Implications of Genre

After analysing the EPR text as dialogic and as a genre (Bakhtin et al., 2010), our findings suggest that, in the EPR, the transition to death involves potential tensions. Sometimes, the voices are aligned with previous notes on the case, and phrases such as “going to sleep” appear as a logical conclusion to the course of dying described. However, sometimes the notes are *not* attuned to one another, and the phrase “going to sleep” stands in contrast to the previous notes. Here, a tension arises between the more clinical, matter-of-fact documentation and a voice describing death in lay terms that corresponds with the vision of a good death. We believe that providing a sanitised or embellished version of death may, in some cases, lead to a lopsided version of the death narrative, as in the news story cited above (Rostad, 2020). In this, we also recognise the frequent use of euphemisms for death. “A euphemism is used as an alternative to a dispreferred expression, in order to avoid possible loss of face, either one’s own face or, through giving offense, that of the audience, or of some third party” (Allan & Burridge, 1991, p. 11). Here, euphemising is a strategy that softens language to preserve social harmony (Jamet, 2012), which concurs with the notion of *death brokering*, understood as the way medical authorities help to render the death culturally acceptable (Timmermans, 2005). Related to the news story, for the writer, the use of “going to sleep” is an unproblematic way of writing “dying”, but for the reader, sleep may imply a peacefulness that they did not witness, thus showing the complex way in which words carry different values depending on the context in which a text is written and readers’ position toward the (con)text (Dentith, 2004). In addition to providing the reader a job well-done, this way of documenting death contributes to sustaining and reproducing a picture of death as an unproblematic event.

## Conclusion

We argue that there are limitations and challenges involved in documenting the transition to death in the EPR. Our analysis shows that the EPR genre, when documenting the transition to death, is ambiguous and unsettled because no matter what happened in the time leading up to death, we see that death is presented as biomedical, uneventful and/or good. From this, two main ethical implications follow: first, the risk of communicating a story that the involved parties (i.e., family) perceive as false or skewed and, second, the fact that an unnuanced description of dying contributes to silencing potential reflections on what might have been otherwise. Compared with our findings on the transition from curative to palliative care (Hov et al., 2020a), the language of the transition to death is more conclusive and less open than that of the negotiation of a turning point, making the text less nuanced. We argue that a genre more tolerant of nuances could contribute to greater openness about patients’ experiences and the quality of care. In doing so, the texts’ ethical responsibility to the subject (i.e., the patient) would be better served, which would, in turn, challenge the established social and ideological norms of the documentation of death for the better.
